# Neochlorogenic acid: an anti-HIV active compound identified by screening of Cortex Mori [*Morus Alba* L. (Moraceae)]

**DOI:** 10.1080/13880209.2021.1995005

**Published:** 2021-10-29

**Authors:** Jing Li, Lu Dou, Shuangfeng Chen, Honghao Zhou, Fangzheng Mou

**Affiliations:** a Department of Central Laboratory, Chongqing University Three Gorges Hospital, Chongqing, China; b College of Life Sciences, Chongqing Medical University, Yuzhong, China; c The Center of Clinical Research of Endocrinology and Metabolic diseases in Chongqing and Department of Endocrinology, Chongqing University Three Gorges Hospital, Chongqing, China; d Internal Medicine of Traditional Chinese Medicine, Chongqing University Three Gorges Hospital, Wanzhou, China

**Keywords:** Traditional Chinese medicine, reverse transcriptase, enrichment analysis

## Abstract

**Context:**

Chinese herbs such as Cortex Mori [*Morus alba* L. (Moraceae)] may inhibit human immunodeficiency virus (HIV), but active compounds are unknown.

**Objective:**

Screening of Cortex Mori and other herbs for anti-HIV active compounds.

**Materials and methods:**

HIV-1 virus (multiplicity of infection: 20), and herbs (dissolved in dimethyl sulfoxide, working concentrations: 10, 1, and 0.1 mg/mL) such as Cortex Mori, etc., were added to 786-O cells (10^5^ cell/well). Zidovudine was used as a positive control. Cell survival and viral inhibition rates were measured. The herb that was the closest inactivity to zidovudine was screened. Mass spectrometry identified the active compounds in herbs (mobile phase: 0.05% formic acid aqueous solution and acetonitrile, gradient elution, detection wavelength: 210 nm). The effect of the compounds on reverse transcriptase (RT) products were evaluated by real-time PCR. Gene enrichment was used to analyse underlying mechanisms.

**Results:**

With a dose of 1 mg/mL of Cortex Mori, the cell survival rate (57.94%) and viral inhibition rate (74.95%) were closest to the effect of zidovudine (87.87%, 79.81%, respectively). Neochlorogenic acid, one of the active ingredients, was identified by mass spectrometry in Cortex Mori. PCR discovery total RT products of neochlorogenic acid group (mean relative gene expression: 6.01) significantly inhibited (control: 35.42, *p* < 0.0001). Enrichment analysis showed that neochlorogenic acid may act on haemopoietic cell kinase, epidermal growth factor receptor, sarcoma, etc., thus inhibiting HIV-1 infection.

**Conclusions:**

For people of low socioeconomic status affected by HIV, Chinese medicine (such as Cortex Mori) has many advantages: it is inexpensive and does not easily produce resistance. Drugs based on active ingredients may be developed and could have important value.

## Introduction

Acquired immune deficiency syndrome (AIDS) is an infectious disease characterised by an injured systemic immune system due to human immunodeficiency virus (HIV) infection (Lu et al. 2018; Seydi et al. 2018). Reverse transcriptase (RT), integrase (IN), and protease (PR) enzymes are essential for three key steps during HIV infection and nucleic acid replication and are also the main targets of HIV drug treatments (Andrabi et al. 2014; Laskey and Siliciano 2014). Recently, the search for new drug targets has been an important trend in HIV drug developmental studies, and RT inhibitors are a hotspot in the development of anti-HIV drugs (Wang et al. 2020). Since HIV RT is not a high-fidelity DNA polymerase and lacks proofreading function, it will cause increased mutation rates in HIV during the replication process. Therefore, the emergence of drug-resistant viruses is inevitable.

Chinese medicines have been used in the treatment of HIV for many years in China. Compared with synthetic compounds, natural compounds extracted from Chinese herbal medicines are characterised by good biological compatibility, relatively low toxicity, and improved immunity. Traditional Chinese herbal medicine may allow for the development of new anti-HIV drugs with low toxicity and high efficacy (Chu and Liu 2011; Harvey et al. 2015).

Chinese herbal medicine is a vital part of traditional Chinese medicine (TCM) and has been used as a treatment technique since its inception in ancient China. Recently, many types of Chinese herbal medicines with different degrees of antiviral activity have been reported (Wan et al. 2020; Yu et al. 2020), including *Glycyrrhiza uralensis* Fischer (Leguminosae) (rhizome) (Wan et al. 2020), *Reynoutria japonica* Houtt (Polygonaceae) (root) (Johnston 1990), *Nepeta cataria* Linn (Labiatae) (stem and leaf) (Johnston 1990), *Lithospermum erythrorhizon* Sieb. et Zucc (Boraginaceae) (root) (Chen et al. 1997), *Sophora flavescens* Alt (Leguminosae) (root) (Chen et al. 1997), *Cinnamomum cassia* Presl (Lauraceae) (bark) (Dai et al. 2012), *Euchresta japonica* Hook. f. ex Regel (Leguminosae) (root) (Sun et al. 2015), and Cortex Mori [*Morus alba* L. (Moraceae)] (bark) (Lee et al. 2007). In this study, eight types of Chinese medicines were selected for study, and their anti-HIV activities were preliminarily evaluated. Chinese medicines with definite HIV inhibitory effects were screened from the eight types of Chinese medicines, and their natural compounds were used for HIV inhibition experiments. Furthermore, their functions and mechanisms were explored to determine the active monomeric compounds in the Chinese medicines that showed targeted inhibition of HIV-1 RT. The study results should provide a theoretical and experimental basis for the drug design, structural modification, and development of a new generation of HIV-RT inhibitors.

## Materials and methods

### Cell line culture

Human renal carcinoma 786-O cells and human embryonic kidney 293 T cells were purchased from Beina Chuanglian Biotechnology Institute (Beijing, China). Cells were stored in Roswell Park Memorial Institute (RPMI) 1640 medium (Gibco, Suzhou, China) or Dulbecco’s modified Eagle’s medium (DMEM) (Gibco, Suzhou, China) and cultured in media containing 10% foetal bovine serum (FBS) at 37 °C in 5% CO_2_.

### Lentivirus packaging

pHIV-lus-zsGreen plasmids were transfected into *DH5α* competent cells and the cell suspension was smeared on LB agar plates (LB medium containing 10 g/L tryptone, 5 g/L yeast extract, 10 g/L NaCl, 15 g/L agar, and 10 μg/mL ampicillin), and bacterial plaques were screened. All operations were performed according to the instructions of the EndoFree Plasmid Maxi Kit (QIAGEN, Dusseldorf, Germany) to obtain many high-purity endotoxin-free plasmids, which were stored at −20 °C. The bacterial solution and glycerine were mixed at a ratio of 1:1 and then stored at −80 °C. The primer was designed in the promoter region of the plasmids, the extracted plasmids were sent to the Beijing Genomics Institute for sequencing, and DNAMAN 6.0 software was used to compare the sequencing results with the reference sequence provided by Addgene (http://www.addgene.org/).

The mixture solutions containing the two plasmids, pHIV-Lus-ZsGreen and 2nd Generation Packaging Mix, were added into a serum-free and double-antibody-free DMEM medium at a ratio of 1:1. Further, the same procedure was performed for the DNAfectin^TM^ Plus Transfection Reagent, and both mixture solutions were incubated for 5 min, respectively. Then, both solutions were co-incubated for 30 min and added to 293 T cells. Subsequently, the cells were incubated at 37 °C in 5% CO_2_ for 6–8 h. Thereafter, the medium was replaced with DMEM complete medium containing 10% FBS. After 48 h of transfection, the expression of green fluorescence proteins in the cells was observed under a fluorescence microscope. The media were collected if the expression of green fluorescence proteins was observed.

The supernatant was collected and centrifuged at 10,000 rpm for 40 min at 4 °C. Then, the supernatant was discarded and the sediment was added to a 0.5 mL medium. The titre of the viruses was determined according to the procedure provided by the qPCR Lentivirus Titration (Titre) Kit; subsequently, the lentiviruses were centrifuged and concentrated to achieve a final titre with an order of magnitude of 10^8^ TU/mL and stored at −80 °C after sub-packaging. The treated viruses were added onto the 786-O cells cultured in a 6-well plate at a volume of 5 μL/well. Cells were then incubated with polybrene (final concentration: 0.8 µg/mL) at 37 °C in 5% CO_2_ for 48 h and the expression level of green fluorescence protein was analysed.

### Detection of cell survival rate and virus inhibition rate

The eight Chinese medicines (*G. uralensis* rhizome, *R. japonica* root, *N. cataria* stem, *L. erythrorhizon* root, *S. flavescens* root, *C. cassia* bark, *E. japonica* root, and Cortex Mori) were provided by the Department of Internal Medicine of Traditional Chinese Medicine at Chongqing University Three Gorges Hospital and were identified by Fangzheng Mou, director of the Department of Internal Medicine of Traditional Chinese Medicine. A total of 200 mg of each granule preparation of the eight medicines was accurately weighed. A liquid nitrogen grinder (40 mesh) was used to grind the granule preparations into powders. The powders were dissolved and mixed with 200 μL dimethyl sulfoxide (DMSO), then treated with ultrasound at room temperature for 30–60 min. The extract solution was cooled to room temperature, and three working concentration gradients were prepared for each drug: 10, 1, and 0.1 mg/mL.

Human renal carcinoma 786-O cells were seeded onto transparent 96-well plates (10^5^ cell/well) and then incubated with the Chinese medicine solutions at the above-mentioned concentrations and HIV-1 viruses (multiplicity of infection = 20) and polybrene (working concentration: 0.8 µg/mL; same below). The cells were further cultured at 37 °C in 5% CO_2_ for 48 h, and subsequently cultured with 10 μL Cell Counting Kit-8 (CCK8) for another 2 h. The cell survival rate was then detected.

Additionally, cells were seeded onto white 96-well plates and the cells were incubated with the eight Chinese medicine solutions at the above-mentioned concentrations. The cells were further incubated at 37 °C in 5% CO_2_ for 1 h and co-cultured with viruses. Subsequently, the cells were incubated with polybrene and the cells continued to be cultured for another 48 h. Next, luciferase substrate was added to the cells and shaken gently in the dark for 15 min.

The luciferase luminescence signals were detected by a microplate reader, and the inhibition rates of the medicinal compositions on lentivirus infection were calculated.

Cell survival rate=[(As−Ab)/(Ac−Ab)]×100%.

Virus inhibition rate=[(As−Ab)/(Ac−Ab)]×100%.

As: Experimental well (the well contained medicinal compositions, cells, and culture medium)

Ac: Negative control well (the well did not contain any medicinal compositions, only cells and culture medium)

Ab: Blank control well (the well did not contain any medicinal compositions, only culture medium).

In the subsequent steps, Chinese medicine granules associated with a high cell survival rate or high cell inhibition rate were selected as candidate medicines for chemical composition analysis.

### Real-time quantitative PCR analysis of HIV-1 DNA

To further determine the mechanism of inhibition on HIV infection in the early stage, the real-time quantitative PCR (qPCR) probe method was used to detect changes in the expression levels of HIV DNA products during HIV-1 cell infection. The primers and probes were designed according to previous studies (King and Attardi 1989; Butler et al. 2001; Brussel and Sonigo 2003; Bacman and Moraes 2007). Mitochondrial DNA was selected as the internal reference, and the primer information is as shown in Table 1 (Butler et al. 2001; Brussel and Sonigo 2003).

**Table 1.** RT-PCR primers and probes.

Target DNA	Primer or probe	Name and sequence
Total RT (R/U5)	Early RT forward	MH535: 5′-AACTAGGGAACCCACTGCTTAAG-3′
	Early RT reverse	Early 2: 5′-CTGCTAGAGATTTTCCACAC-3′
	Early RT probe	MH603: 5′-(FAM)-ACACTACTTGAAGCACTCAAGGCAAGCTTT-(BHQ)-3′
Late RT (flank PBS)	Late RT forward	MH531: 5′-TGTGTGCCCGTCTGTTGTGT-3′
	Late RT reverse	MH532: 5′-GAGTCCTGCGTCGAGAGAGC-3′
	Late RT probe	LRT-P: 5′-(FAM)-CAGTGGCGCCCGAACAGGGA-(BHQ)-3′
Integrated DNA (Alu-LTR)	First-round Alu-LTR PCR	
	LTR forward	GSTLTR: 5′-AGACAGATAGGGCCGTTAAACTAGGGAACCCACTGCTTAAG-3′
	Alu reverse	Alu 2: 5′-GCCTCCCAAAGTGCTGGGATTACAG-3′
	Second round real-time PCR	
	GST forward	GST: 5′-AGACAGATAGGGCCGTTAAAC-3′
	LTR reverse	Early 2: 5′-CTGCTAGAGATTTTCCACAC-3′
	Probe	MH603: 5′-(FAM)-ACACTACTTGAAGCACTCAAGGCAAGCTTT-(BHQ)-3′
mtDNA (10,620–10,710)	mtDNA forward	MH533: 5′-ACCCACTCCCTCTTAGCCAATATT-3′
	mtDNA reverse	MH534: 5′-GTAGGGCTAGGCCCACCG-3′
	mtDNA probe	mito-probe: 5′-(FAM)-CTAGTCTTTGCCGCCTGCGAAGCA-(BHQ)-3′

Chinese medicines and the positive control medicine, zidovudine (AZT), were both dissolved in DMSO. The initial concentration of Chinese medicines was determined based on the results from a previous step. The initial concentration of the AZT was 100 μg/mL.

Human renal carcinoma 786-O cells were cultured in 6-well plates, and the cells + AZT, the cells + lentivirus, and untreated cells were assigned to the positive control group, negative control group, and blank control group, respectively. The cells were initially incubated with medicine supplementation for 1 h and then were incubated with lentivirus and polybrene at 37 °C in 5% CO_2_ for 8 h or 24 h, respectively. (Note: The reaction time of products detected by different probes is different). The cells in the 6-well plates were subsequently washed gently 8–10 times with normal saline to remove the remaining drugs and viruses. Subsequently, the cells were digested with pancreatin and washed thrice with normal saline. DNA was extracted from the cells according to the method provided by the DNeasy Blood and Tissue (QIAGEN, Dusseldorf, Germany) kit, and the DNA concentration was measured. The isolated DNA was stored at −20 °C until further use. Three parallel experiments were performed for each group.

Detection of total RT products and late RT products: The primers and probes in Table 1 were used to detect the expressions of total RT products and late RT products at 8 h after incubation with lentivirus and medicine supplementation. The reaction system contained 1 µL DNA template (100 ng/µL), 0.5 µL upstream and downstream primers (900 mmol), 0.5 µL probe, and 10 µL qPCR detection reagent; some water was added to reach a final volume of 20 µL, and three replicate wells were established. The reaction procedure was 55 °C for 3 min, 95 °C for 5 s, and 60 °C for 30 s for 40 cycles.

Detection of integrated DNA: DNA products obtained after 24 h incubation with lentivirus and medicine supplementation were used for this step. Firstly, the primers in Table 1 were used to perform Alu-LTR PCR; the reaction system contained 1 µL DNA template (100 ng/µL), 0.5 µL upstream and downstream primers (900 mmol), and 10 µL High Fidelity PCR Enzyme Mix; water was added to make a final volume of 20 µL. The reaction procedure was 95 °C for 5 min, 95 °C for 30 s, 60 °C for 30 s, and 72 °C for 3 min for 16 cycles; and then 72 °C for 5 min. The products were stored at 16 °C until use. Further, 1 µL of Alu-LTR PCR product was used for qPCR detection using the same method as previously described.

### Separation and identification of natural compound constituents contained by liquid mass spectrometry

Based on the experimental results obtained from the survival rate and viral inhibition rate analyses of the lentivirus-transfected cells treated with eight Chinese medicines, we chose the best drug for further experiments. According to previously published studies (Feng et al. 2013; Zhiyong Chen et al. 2018; Guo 2019), select monomers that may have anti-HIV effects. These monomers were analysed and detected by liquid mass spectrometry. A total of 50 mg of each Chinese medicine was weighed and dissolved in 50 μL DMSO (as above), and then treated by ultrasound at room temperature for 30 min and filtered through a 0.22 μm organic membrane. A 5 μL injection sample was taken for each LC/MS analysis. The conditions for liquid mass spectrometry were as follows:

Chromatographic column: Shim-pack VP-ODS18 (250 mm × 2.0 mm, 5 μm); mobile phase: 0.05% formic acid aqueous solution (A), acetonitrile (B), gradient elution (0–5 min, 9% B; 5–22 min, 9–22% B; 22–40 min, 22–65% B; 40–60 min, 65–95% B; 60–65 min, 95% B); detection wavelength: 210 nm; flow rate: 0.3 mL/min; column temperature: 30 °C, and injection volume: 5 μL.

Mass spectrometry conditions: ionisation mode: ESI (±); atomising gas flow rate: 3.0 L/min; drying gas flow rate: 10 L/min; heating gas flow rate: 10 L/min; interface temperature; 450 °C; DL temperature: 300 °C; and heating module temperature: 400 °C.

Afterward, real-time quantitative PCR analysis of the identified 10 monomers (working concentration: 1 mg/mL) on HIV-1 DNA was performed using the same method, as shown above.

### Screening of monomer targets

We used PubChem (https://pubchem.ncbi.nlm.nih.gov/) and Discovery Studio 2020 Client to obtain the monomer structure, uploaded the structural formula to PharmMapper (http://www.lilab-ecust.cn/pharmmapper/) (Wang et al. 2017), target selection ‘Human Protein Targets Only (v2010, 2241)’, and uploaded the predicted protein target to UniProt (https://www.uniprot.org/) with Normalised Fit Score > 0.7 to obtain the gene targets. We uploaded the obtained gene targets to Metascape (https://metascape.org/gp/index.html#/main/step1) (Zhou et al. 2019) for Gene Ontology (GO) enrichment analysis and Kyoto Encyclopaedia of Genes and Genomes (KEGG) pathway analysis and uploaded the enrichment analysis GO results to a bioinformatics online tool (http://www.bioinformatics.com.cn/) for visualisation. We then uploaded the obtained gene targets to String (https://string-db.org/) to construct a protein-protein interaction (PPI) network and used Cytoscape 3.7.2 to perform further processing of the results.

### Analysis of potential targets of monomers acting on HIV

We used PathCard (https://PathCards.genecards.org/) (Belinky et al. 2015) to obtain all gene names for HIV-related pathways and used a bioinformatics online tool (http://www.bioinformatics.com.cn/) to obtain the intersection of HIV-related genes and the target of monomer. The results contained possible targets of monomer and HIV. We then performed enrichment analysis and PPI analysis on the target again.

### Statistical analysis

Results are presented as the mean ± standard error of the mean (SEM) for the sample number of each group. One-way analysis of variance (ANOVA) was used to evaluate the significance between multiple groups. Tukey’s multiple comparisons test was used to compare the mean values of two specific groups in GraphPad Prism 8.0.1, and *p* < 0.05 was considered significant.

## Results

### HIV packaging

**Figure 1.**
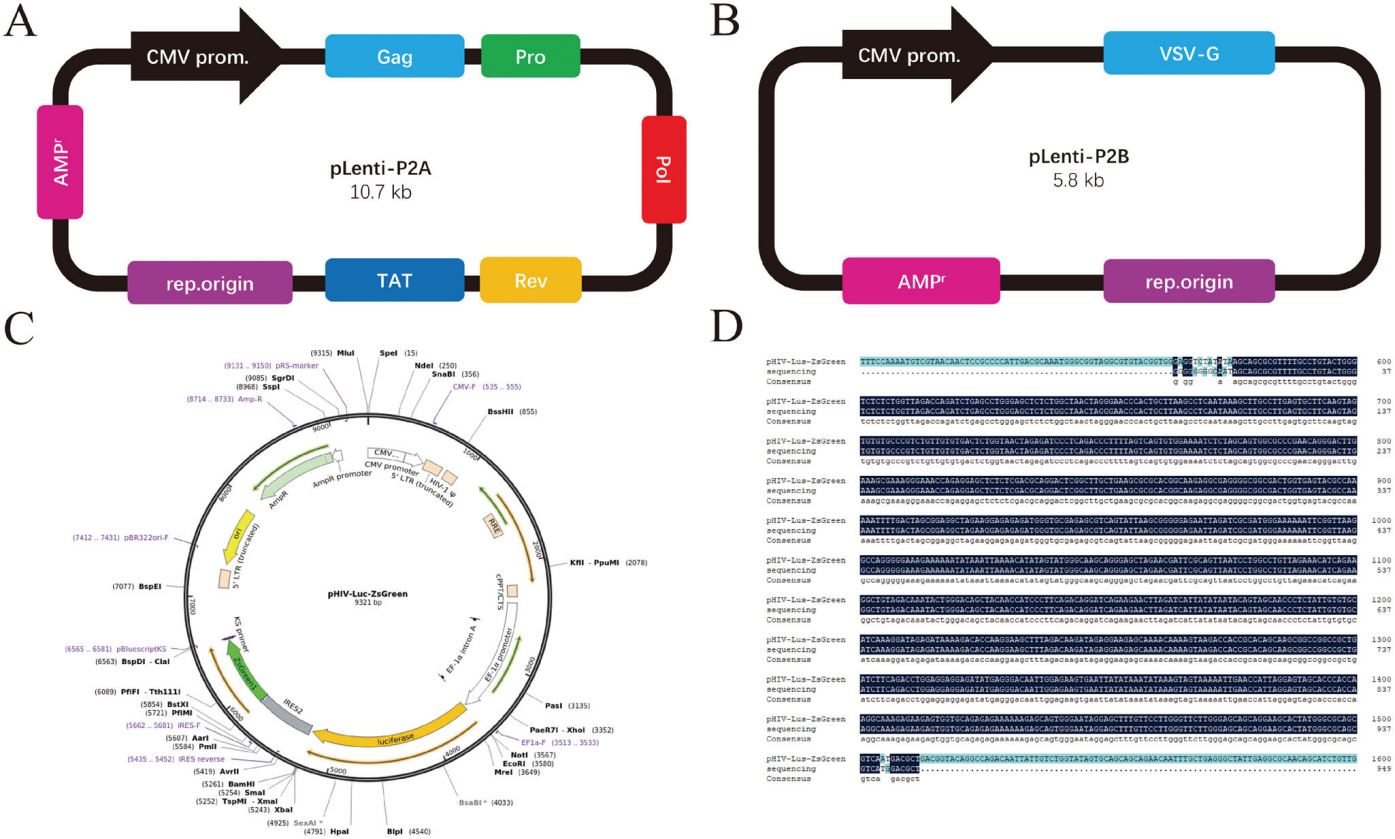
Vector map and sequencing results. (A) pLenti-P2A Vector. (B) pLenti-P2B Vector. (C) pHIV-lus-ZsGreen Vector. (D) Sequence alignment results.

The pHIV-Lus-ZsGreen plasmid was sequenced and compared, and the sequence was consistent with the reference sequence, suggesting the vector was correct and subsequent experiments could be performed (Figure 1).

### Lentivirus-infected cells

**Figure 2.**
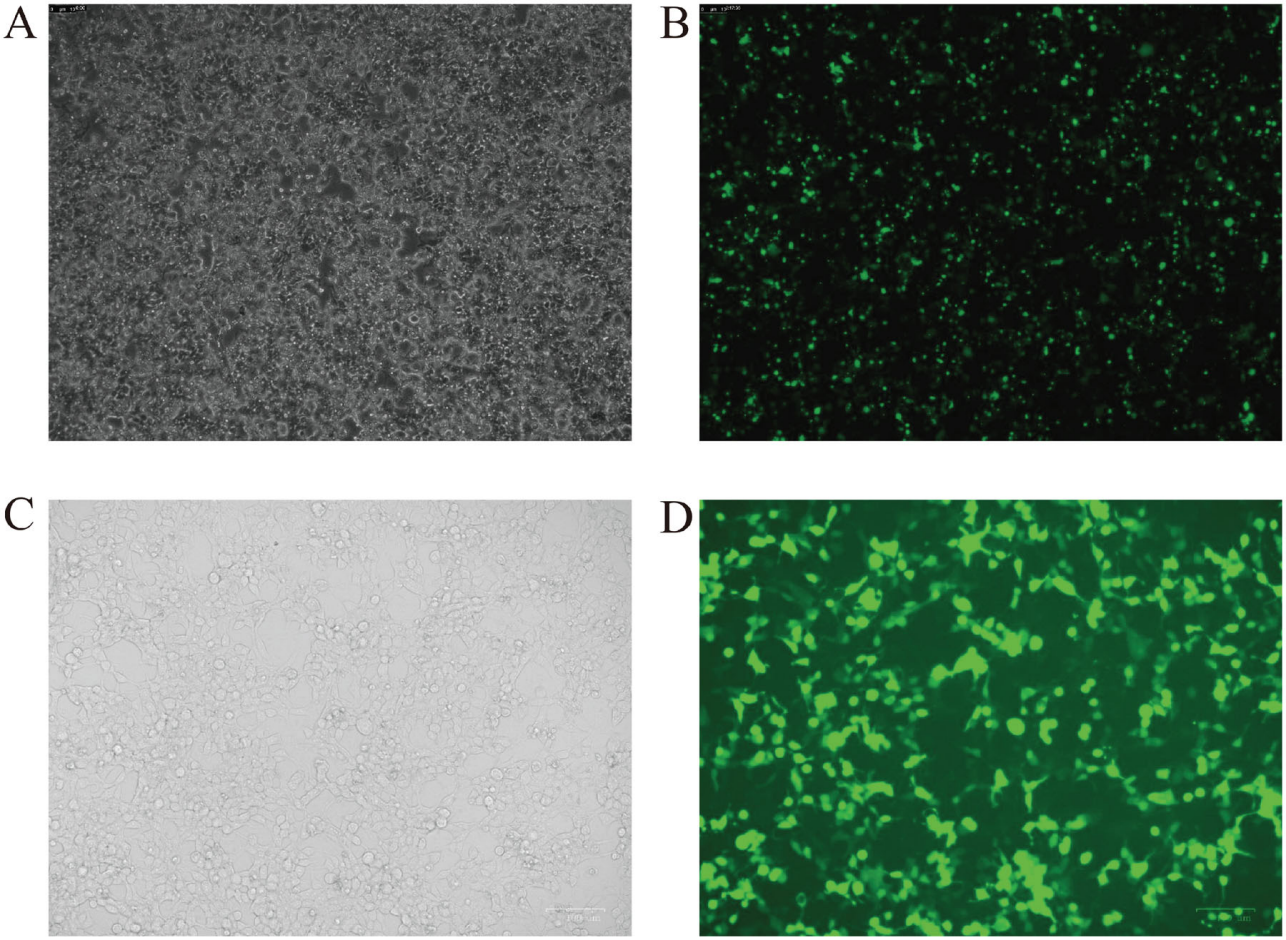
Plasmid transfection and viral packaging. (A,B) Transfection of plasmids into 293 T cells, (C,D) 786-O cells infected by packaged viruses.

Fluorescence microscopy was performed at 48 h after co-transfection with three plasmids into 293 T cells. The results indicated high expression levels of green fluorescent proteins (GFP), suggesting that the plasmid was successfully transfected into the cells (Figure 2(A,B)). The supernatant was collected at 48 h after transfection and added into 786-O cells. The cells expressed green fluorescence 48 h later (Figure 2(C,D)), suggesting that the viruses were successfully packaged. After being concentrated, the titre of the viruses was detected according to the method provided by the virus detection kit, and the viruses were packed separately and stored at −80 °C.

### Preliminary screening results of eight Chinese medicines

**Figure 3.**
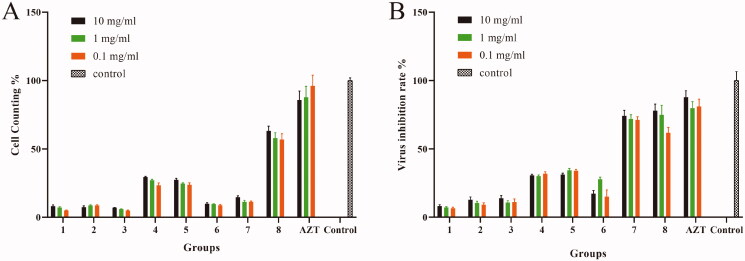
Screening results of eight Chinese medicinal compositions. (A) Effects of eight Chinese medicinal compositions on cell survival rates. (B) Effects of eight Chinese medicinal compositions on viral inhibition rates. 1: *G. uralensis*; 2: *R. japonica*; 3: *N. cataria*; 4: *L. erythrorhizon*; 5: *S. flavescens*; 6: *C. cassia*; 7: *E. japonica*; 8: Cortex Mori.

The cell survival rate and virus inhibition rate were detected and compared among eight groups treated with Chinese medicines. The cell survival rate in the Cortex Mori group (the means of the high to low concentration groups were 63.28%, 57.94%, 56.94%; same below) was the highest (Figure 3(A)). The HIV inhibition rates of the Cortex Mori group (77.94%, 74.95%, and 61.75%) and *N. cataria* group (74.24%, 71.91%, and 71.26%) were higher than those of the other six Chinese medicine groups (Figure 3(B)). Thus, Cortex Mori was selected to further study its action mechanism. Since the experiment results at 10 mg/mL and 1 mg/mL were similar, 1 mg/mL was selected as the initial concentration of Cortex Mori in the follow-up study.

### Effects of Cortex Mori on the products of HIV RT at different stages

**Figure 4.**
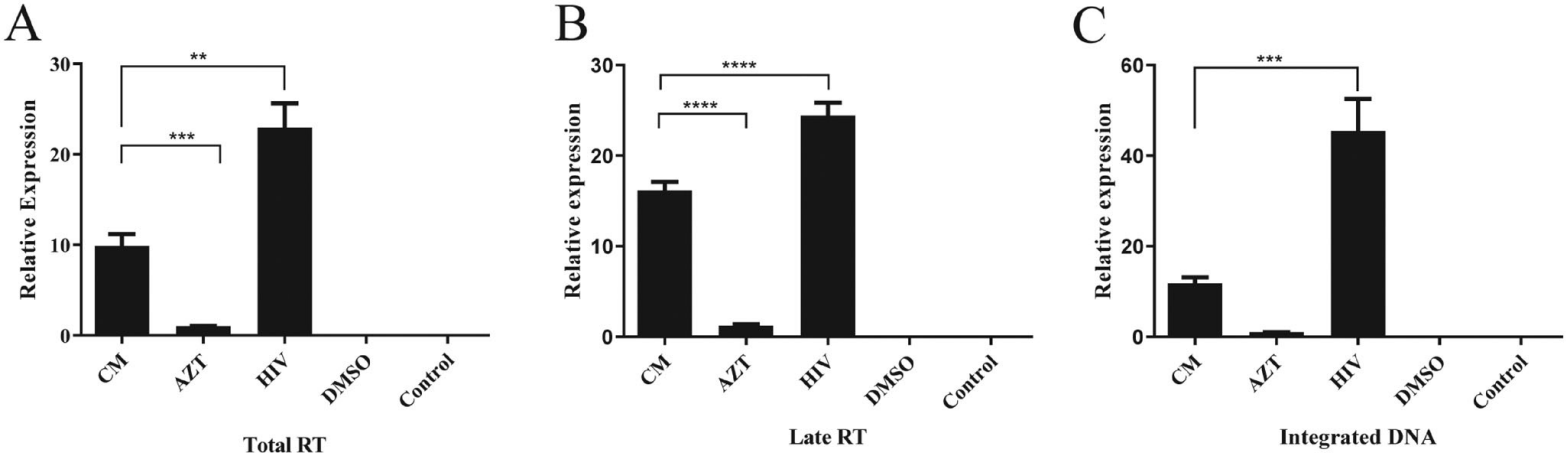
Effects of Cortex Mori on the expression of RT enzyme products at different stages. (A) Effects of Cortex Mori on the expression of total RT enzyme products. (B) Effects of Cortex Mori on the expression of late RT enzyme products. (C) Effects of Cortex Mori on the expression of integrated DNA enzyme products. CM group: DMSO, HIV, and Cortex Mori were added to the cells; AZT group: only AZT and DMSO were added to the cells; HIV group: only HIV and DMSO were added to the cells; DMSO group: only DMSO was added to the cells; control group: the cells did not receive any medicinal treatments. Data are expressed as the mean ± SEM. **p < 0.01, ***p < 0.001, ****p < 0.0001.

When treated with Cortex Mori (1 mg/mL), the total RT group, late RT group, and integrated DNA group (each group’s relative gene expression mean values were 9.88, 16.16, and 11.83, respectively; same below) compared with the HIV group (the mean values were 22.94, 24.45, and 45.43, respectively) were significantly reduced (all *p* < 0.05). Those of the Cortex Mori group were higher than those of the AZT control group (1.00, 1.21, and 1.00, respectively; all *p* < 0.01) (Figure 4(A–C)). The results showed that Cortex Mori had a significant inhibitory effect on the HIV-1 RT enzyme, but the effect was not as favourable as that of AZT.

### Chemical monomers in Cortex Mori detected by liquid mass spectrometry

**Figure 5.**
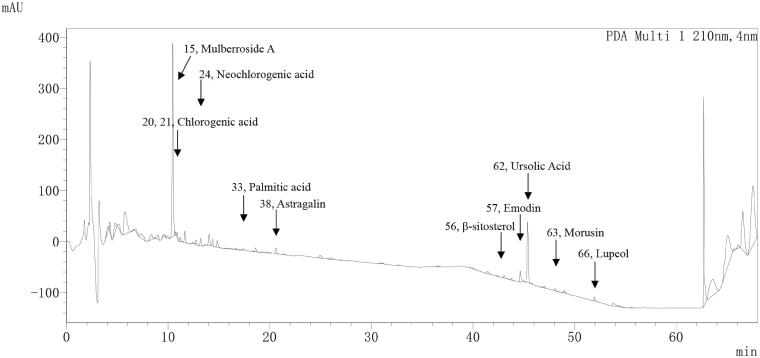
Chromatogram of liquid phase mass spectrum peaks for separation and identification of Cortex Mori granules.

To identify the chemical composition of Cortex Mori granules and to further clarify the active components of natural compound monomers contained in Cortex Mori granules, and to identify structural characteristics for the development of new antiviral drugs, according to previously published studies (Feng et al. 2013; Zhiyong Chen et al. 2018; Guo 2019), 10 Chinese medicine monomers in Cortex Mori were selected, and 10 standard compounds were identified in Cortex Mori granules by liquid-phase mass spectrometry gradient elution and mass spectrometry analysis, including mulberroside A (peak number: 15; same below), chlorogenic acid (20, 21), neochlorogenic acid (24), palmitic acid (33), astragalin (38), β-sitosterol (56), emodin (57), ursolic acid (62), morusin (63) and lupeol (66) (Figure 5, Table 2).

**Table 2.** Chromatographic peak parameters corresponding to the chromatograms of 10 Chinese medicine monomers contained in *Mori Cortex*.

Item	Peak number	Name	Retention time	Area	High
1	15	Mulberroside A	10.457	2,824,281	378,595
2	20	Chlorogenic acid	11.658	178,434	22,963
3	21	Chlorogenic acid	12.213	3288	496
4	24	Neochlorogenic acid	13.212	116,590	15,164
5	33	Palmitic acid	17.393	32,853	3500
6	38	Astragalin	19.988	20,374	1741
7	56	β-Sitosterol	43.795	21,922	3192
8	57	Emodin	44.436	1623	393
9	62	Ursolic Acid	47.045	24,769	3121
10	63	Morusin	48.084	48,665	5922
11	66	Lupeol	51.958	50,306	7128

### Effects of 10 monomers contained in Cortex Mori on the products of RT

Fluorescent PCR and probe-based quantitative methods were used to further verify the inhibitory effect and action mechanism of the 10 natural compound molecular monomers contained in Cortex Mori on HIV-1 RT, to obtain new natural compound monomer molecules with effective anti-HIV effects.

**Figure 6.**
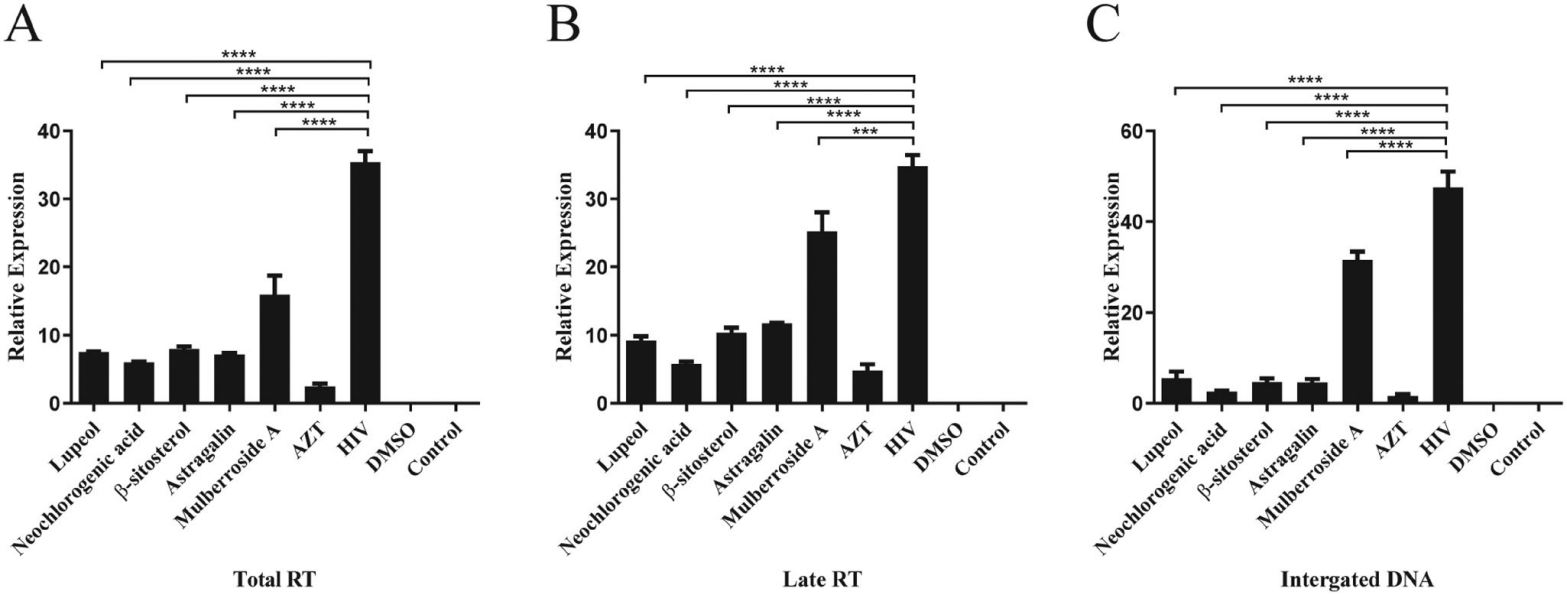
Effects of five active monomer compounds in Cortex Mori on the expression of products at different stages of HIV infection. (A) Effects of five active monomer compounds in Cortex Mori on the expression of total RT enzyme products. (B) Effects of five active monomer compounds in Cortex Mori on the expression of late RT enzyme products. (C) Effects of five active monomer compounds in Cortex Mori on the expression of integrated DNA products. Data are expressed as the mean ± SEM. ***p < 0.001, ****p < 0.0001.

Ten types of Chinese medicine monomers were added into the cells, and then DNA products at different periods were collected. The results showed that five monomeric compounds, such as emodin, ursolic acid, morusin, chlorogenic acid, and astragalin had greater cytotoxicity at a concentration of 1 mg/mL, and the cell survival rate was low (data not published), which led to difficulty in extracting DNA to complete the follow-up test. Therefore, we selected the DNA products of the other five compounds, such as lupeol, neochlorogenic acid, β-sitosterol, palmitic acid, and mulberroside A for quantitative analysis, and the changes in RT products at each stage were determined by PCR assays (Figure 6(A–C)). The results showed that lupeol, neochlorogenic acid, β-sitosterol, palmitic acid, and mulberroside A could inhibit the activity of the RT enzymes to some extent. neochlorogenic acid and AZT had the most similar inhibitory effect (the most inhibited effect in 10 monomer) (Figure 6(A–C)). Compared with the HIV group three stages products (mean of the related expression level of each group: 35.42, 34.78, 45.57), neochlorogenic acid group (6.01, 5.76, 2.53) were significantly decreased (all *p* < 0.0001).

### Screening of neochlorogenic acid targets

**Figure 7.**
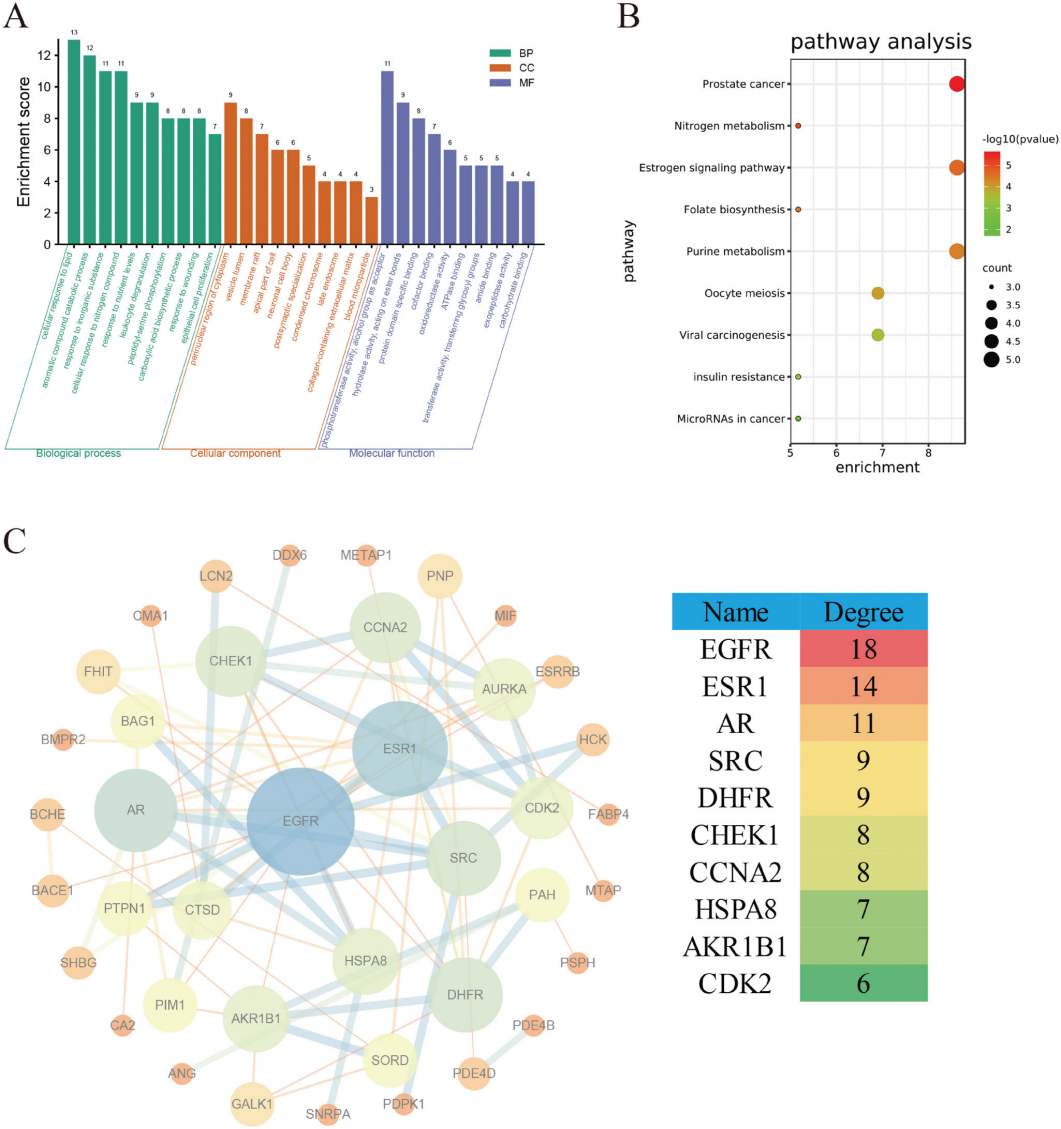
Analysis of the target of neochlorogenic acid. (A) GO enrichment analysis. (B) Pathway analysis. (C) PPI analysis.

The PubChem ID of neochlorogenic acid is 5280633, the molecular formula is C16H18O9, and the molecular weight is 354.31 g/mol. A total of 58 protein targets with neochlorogenic acid (normalised fit score > 0.7) were obtained. GO enrichment analysis revealed that these targets mainly involve cellular reactions (such as cellular response to lipid and response to inorganic substance), synthetic or metabolic processes (such as aromatic compound catabolic process, carboxylic acid biosynthetic process, and other biological processes), and molecular functions (such as phosphotransferase activity, hydrolase activity, oxidoreductase activity, and protein domain specific binding) (Figure 7(A)). The pathway analysis showed that the main pathways were prostate cancer, oestrogen signalling pathway, nitrogen metabolism, and other signalling pathways (Figure 7(B)). With PPI analysis, after removal of outliers, a PPI network with 40 nodes and 82 edges was obtained, with an average node degree of 4.2; the largest node degrees were for EGFR, ESR1, and AR (node degrees of 18, 14, and 11, respectively) (Figure 7(C)).

### Target analysis of neochlorogenic acid on HIV

**Figure 8.**
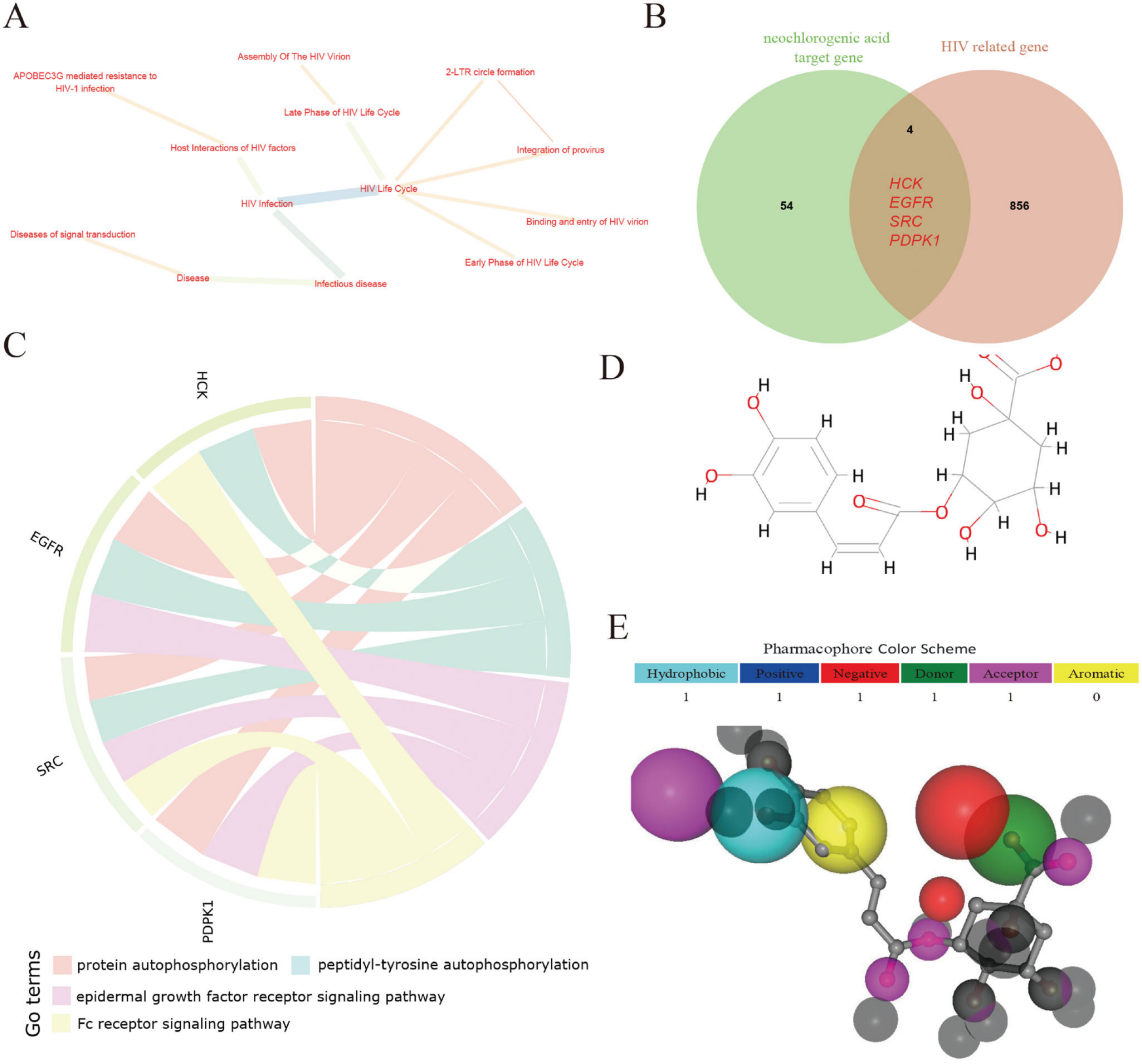
Target analysis of neochlorogenic acid acting on HIV. (A) HIV-related pathway. (B) The intersection of HIV-related genes and new chlorogenic acid targets. (C) Intersection gene enrichment results. (D) The structural formula of neochlorogenic acid. (E) Combination model of neochlorogenic acid and 1MU2.

A total of 860 genes in 13 pathways were obtained using PathCard, among which the pathways with the highest node degrees were for HIV life cycle and HIV infection (Figure 8(A)). After crossing with the neochlorogenic acid target, four genes were obtained: haemopoietic cell kinase (*HCK*), epidermal growth factor receptor (*EGFR*), sarcoma (*SRC*), and 3-phosphoinositide dependent protein kinase 1 (*PDPK1*) (Figure 8(B)). The enrichment analysis results of these four genes showed that they were mainly concentrated in protein autophosphorylation, peptidyl-tyrosine autophosphor, epidermal growth factor receptor, and Fc receptor signalling pathway functional categories (Figure 8(C)). According to the structural formula of neochlorogenic acid (Figure 8(D)), we unexpectedly discovered that it can bind HIV type 2 (HIV-2) RT (PDB ID: 1MU2) (Figure 8(E)).

## Discussion

Under the action of viral RT, pre-viral DNA is synthesised with viral RNA as the template. Antiretroviral drugs can be used to treat patients with HIV retrovirus infection. The American National Institutes of Health (NIH) and National Centre for AIDS/STD Control and Prevention (NCAIDS/STD) of the China Centre for Disease Control and Prevention have recommended the use of antiretroviral drugs to treat patients with AIDS-related symptoms. Different types of antiretroviral drugs act on various stages of the HIV reverse transcription process. However, the consumption of complex combinations of current anti-HIV chemical medicines may have serious side effects or the virus may become resistant to the medicines.

Chinese medicines have been widely used to fight viruses for decades in China. Recently, Chinese medicines played a significant role in the treatment of patients with novel coronavirus pneumonia in China. Tens of thousands of patients have recovered and been cured under the intervention of Chinese medicines, proving their safety and effectiveness in antiviral treatments (Luo et al. 2020; Ren et al. 2020; Yang et al. 2020). In general, Chinese medicines have complex components. Many natural compounds from Chinese medicines, such as coumarin, alkaloid, lignin, flavonoids, tannins, and terpenes have been proven to inhibit HIV RT *in vitro* (Li et al. 2020).

Cortex Mori is a common medicinal material in TCM; it has many pharmacological effects, including anti-inflammatory and analgesic, antitussive and anti-asthmatic, diuretic, hypoglycaemic, hypolipidemic, hypotensive, antitumor, and antiviral effects, and it can improve peripheral neuropathy. However, the pharmacological mechanism of Cortex Mori remains unclear (Du et al. 2003; Hou et al. 2018; Yu et al. 2019; Lu et al. 2020). We found that Cortex Mori significantly inhibited the activity of HIV-1 RT enzyme, thus blocking the replication of the virus. In anti-HIV experiments *in vitro*, Shi de Luo et al. (1995) found that 50 types of Chinese medicines, including Cortex Mori, had anti-HIV effects. Cortex Mori is also one of the components of ‘Compound SH’ (An anti-HIV compound of TCM) (Cheng et al. 2015). A clinical study showed that the decline in CD4 cell count was slowed and reversed in AIDS patients taking oral compound SH (Kusum et al. 2004). Such results are consistent with the results of our study, which shows that mulberry bark is a potential anti-HIV drug.

Shi de Luo et al. (1995) identified six components, morusin, kuwanon H, mulberofuran D, mulberofuran K, mulberofuran G, and kuwanon, from Cortex Mori, among which morusin and kuwanon H have been indicated to have anti-HIV activities from *in vitro* experiments. Xue (2009) obtained nine compounds by mass spectrometry, including tritriacontane, hexadecanoic acid, β-sitosterol, betulic acid, oleanolic acid, scopoletin, 4′,5,7-trihydroxy-8-(3,3-dimethylallyl)-flavone, morusignin L, and daucostero, with some anti-HIV effects. In this study, 10 chemical components were identified by mass spectrometry: ursolic acid, emodin, palmitic acid, β-sitosterol, neochlorogenic acid, mulberroside A, chlorogenic acid, astragalin, morusin, and lupeol. We found that neochlorogenic acid in Cortex Mori had the best pharmacological activity. Combined with findings from previous studies, our results show that mulberry contains a variety of anti-HIV components, and neochlorogenic acid maybe just one of them; therefore, the others require further research. Our results also highlight the multi-component and multi-target characteristics of Chinese medicine.

In our research results, the four genes *HCK*, *EGFR*, *SRC*, and *PDPK1* may be potential targets through which neochlorogenic acid inhibits HIV infection in humans. HCK protein is a member of the Src family of non-receptor tyrosine kinases, which is preferentially expressed in myeloid and B lymphoid haematopoietic cells (Moarefi et al. 1997). Nef is a multifunctional pathogenic protein of HIV-1, and its interaction with the Src tyrosine kinase Hck, which is highly expressed in macrophages, is related to the development of AIDS (Suzu et al. 2005; Hiyoshi et al. 2008). We speculate that neochlorogenic acid may competitively bind HCK protein, thereby inhibiting Nef protein function. Few studies have examined EGFP and HIV, and they have mainly focussed on mutations (Crequit et al. 2016; Walline et al. 2017; Liu et al. 2019). Kaposi sarcoma (KS) is the most common malignant tumour in HIV/AIDS. HIV-related exosomes increase the expression of HIV transactivator (TAR) RNA and EGFR in oral mucosal epithelial cells, thereby promoting Kaposi sarcoma-associated herpesvirus (KSHV) infection (Chen et al. 2020). Lin et al. (2011) found that the phosphorylation level of PDPK1 is closely associated with the expression of p300 protein, which regulates the function of the HIV-1-encoded RNA-binding protein Tat. The above results all show that our prediction results are fairly reliable. In addition, we found that neochlorogenic acid can bind the RT of HIV-2, thus suggesting that neochlorogenic acid may have a favourable inhibitory effect on HIV-2. This result further shows that neochlorogenic acid is a potent inhibitor of HIV-1 virus replication.

In follow-up research, the anti-HIV effects of Cortex Mori or neochlorogenic acid should be further studied through enzyme kinetics, primer extension, band shift experiments, and RNase H activity detection, as well as cell biology, and the possible molecular mechanism of gene expression should be investigated.

In summary, Cortex Mori has anti-HIV effects and lower cytotoxicity, which may be mainly achieved by inhibiting the function of RT enzymes. In addition, HCK, EGFR, SRC, and PDPK1 may be protein targets allowing neochlorogenic acid to inhibit HIV infection in the human body. The precise effect of Cortex Mori on RT needs further study. The existing anti-HIV treatment strategies approved by the FDA are very expensive for more than 90% of HIV-infected patients in developing countries. There have been many studies on the treatment of AIDS with Chinese medicine. It is of great significance to explore TCMs and Chinese medicines to develop affordable drugs with significant effects to prevent and treat AIDS.

## Conclusions

We analysed the anti-HIV effects of eight types of TCMs and found that Cortex Mori inhibits HIV. We further screened and identified 10 compounds from Cortex Mori. Cell experiments indicated that neochlorogenic acid has a good inhibitory effect on the HIV-1 RT enzyme. Neochlorogenic acid may also inhibit HIV through four targets: HCK, EGFR, SRC, and PDPK1. Moreover, neochlorogenic acid may bind HIV-2 RT. Our research can facilitate the development and utilisation of Chinese herbal medicines, and it can serve as a reference for the development of anti-HIV-1 drugs.

## Data Availability

The relevant data have been uploaded as supplementary and can also be obtained from the corresponding author.
